# Parents' experiences of VOICE: A novel support programme in the NICU

**DOI:** 10.1111/nicc.12569

**Published:** 2020-10-29

**Authors:** Agnes van den Hoogen, Rian Eijsermans, Henriette D. L. Ockhuijsen, Floor Jenken, Sabine M. Oude Maatman, Marian J. Jongmans, Lianne Verhage, Janjaap van der Net, Jos M. Latour

**Affiliations:** ^1^ Department of Neonatology, Wilhelmina Children's Hospital University Medical Centre Utrecht Utrecht The Netherlands; ^2^ Centre for Child Development Exercise and Physical Literacy, Wilhelmina Children's Hospital University Medical Centre Utrecht Utrecht The Netherlands; ^3^ Department of Reproductive Medicine and Gynaecology University Medical Centre Utrecht Utrecht The Netherlands; ^4^ Department of Education and Pedagogy Utrecht University Utrecht The Netherlands; ^5^ School of Nursing and Midwifery, Faculty of Health: Medicine, Dentistry and Human Sciences University of Plymouth Plymouth UK

**Keywords:** family support programme, family‐centred care, neonatology, parents, preterm infants

## Abstract

**Background:**

Admission of an infant to a neonatal intensive care unit (NICU) is often a stressful experience for parents and can be associated with feelings of inadequacy to fulfil the desirable parental role. The values, opportunities, integration, control, and evaluation (VOICE) programme was developed to engage parents in care, to decrease stress, and to increase empowerment.

**Aim:**

To explore the experiences of parents regarding involvement in the VOICE programme during admission of their infant to the NICU.

**Design:**

The VOICE programme includes at least five personal structured meetings between parents, nurses, and other health care professionals throughout the pathway from birth, NICU, and follow up. A qualitative design was adopted using semi‐structured interviews. Interviews with 13 parents of 11 infants born at <27 weeks' gestational age were conducted: nine mothers and two couples of father and mother. Thematic analysis was deployed.

**Results:**

The findings have been described in one overarching theme: “parental empowerment.” Parents felt strengthened and were empowered in the development of their role as primary caretaker by the VOICE programme. The parental empowerment theme emerged from four related interpretive themes that were derived: (a) involvement in care, (b) personalized information and communication, (c) transition to a parental role, and (d) emotional support.

**Conclusion:**

The VOICE programme can be a structured approach used to implement family support in a NICU to empower parents to become a partner in the care of their infant and feel confident.

**Relevance to clinical practice:**

This study encourages health care professionals to provide parental support through a structured intervention programme, which contributes to the empowerment of parents in the NICU and encouraged them to participate in care and decision‐making.

**What is known**

- Admission of an infant to a neonatal intensive care unit is a stressful experience for parents.
- Parents experience feelings of inadequacy in fulfilling their parental role.

**What is new**

- Participation and involvement in care and personalized meetings are important factors to support parents in a NICU.
- Parents feel empowered in their parental role when they are informed and encouraged to participate in care and decision‐making.
- The VOICE programme as a parent support intervention contributes to the empowerment of parents in the NICU.

## 1 INTRODUCTION

Admission of an infant to a neonatal intensive care unit (NICU) is often a stressful experience for parents and can be associated with feelings of inadequacy in fulfilling the desirable parental role.^1^, ^2^ Worries about the infant's health, the unfamiliar setting, technology, and monitoring can interrupt normal family functioning and bonding.^2^

To support parent‐infant interaction and the parental role during NICU admission, different programmes have been developed.^3^, ^4^, ^5^, ^6^, ^7^ Complementary to the well‐known Newborn Individualized Development Care en Assessment Program (NIDCAP) and kangaroo care interventions, these programmes support parents based on the principles of family‐centred care (FCC) and family‐integrated care (FIC), with an emphasis on family support and facilitating parents' understanding of their child's developmental and physical care. Melnyk et al describes the Creating Opportunities for Parent Empowerment (COPE) programme including parents of premature infants as standard practice, while O'Brien et al developed the Family Integrated Care model in neonatal intensive care.^3^, ^5^, ^6^, ^8^ The programmes often include parents of premature infants born at <37 weeks of gestational age (GA) and extremely premature infants at <27 weeks of GA.^8^, ^9^, ^10^, ^11^, ^12^, ^13^ Evaluations of these programmes have demonstrated a reduction in parental depression, anxiety, and stress, as well as improved parental empowerment, confidence, and competence.^5^, ^8^, ^13^ However, the previously developed parent support interventions mainly concentrate on the clinical admission period and lack an evaluation component after discharge.

A structured values, opportunities, integration, control and evaluation (VOICE) programme was developed to empower parents of extreme premature infants. The VOICE programme is inclusive throughout the pathways of care of an extreme premature infant from prenatal to the follow‐up period after NICU admission. The VOICE programme is specifically developed to empower parents of extreme premature born infants at <27 weeks of gestation as they might benefit the most from this programme because of their extended length of stay in the NICU.

The aim of this study was to explore the experiences of parents regarding their involvement in the VOICE programme, specifically during the period the infants were admitted to the NICU.

## 2 METHODS

### 2.1 Design

A qualitative research method was adopted with face‐to‐face semi‐structured interviews. The guideline “Consolidated criteria for reporting qualitative research (COREQ): a 32‐item checklist for interviews and focus groups” has been used to report this study.^14^

### 2.2 Setting

The study was conducted in a 24‐bed tertiary NICU in The Netherlands with around 650 annual admissions.

### 2.3 Sample and recruitment

Convenience sampling was used to gain insights on different perspectives. The inclusion criteria were being a parent of an infant born at <27 weeks GA admitted to the NICU and participating in the VOICE programme. Parents were excluded if they were unable to speak Dutch or if their infant had a prognosis of imminent death. Parents were informed about the study by an independent researcher, and at that time, they received both oral and written information. Parents were approached and asked to participate in order of admission of their infant. When parents were willing to participate and gave consent, an appointment for an interview was made.

### 2.4 VOICE Programme

To support and empower parents of preterm infants, a programme was developed called VOICE. The VOICE programme is based on previous research indicating the need for a structured support programme that meet the needs and wishes of parents in the NICU.^15^, ^16^, ^17^ Compared with previously developed FCC interventions to support parents, the novelty of the VOICE programme is that it is designed to support parents throughout the entire pathway, including prenatal, birth, NICU, and follow‐up care.

**FIGURE 1:**
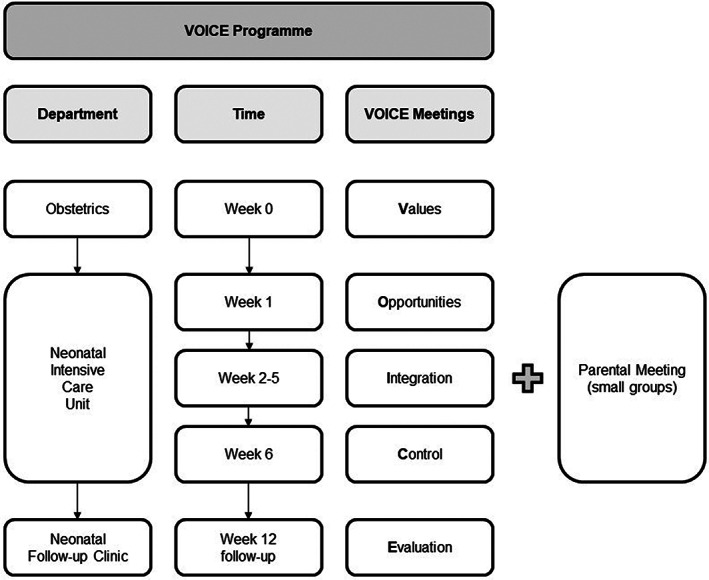
Values, opportunities, integration, control, and evaluation (VOICE) programme and parent meetings

The programme includes at least five structural interdisciplinary‐focussed meetings with parents, before, during, and after an NICU admission, to inform and discuss with parents their role in how to care for their infant and to build a partnership between parents and caregivers. The five VOICE meetings, lasting around 20 to 30 minutes each, are designed with a specific focus (Figure 1). The first meeting is focused on values (V): During an antenatal conversation at the obstetric ward, the focus of this meeting is on preparing the parents for NICU admission and building mutual trust and confidence between parents and health care professionals (nurses, neonatologists, and social workers). The second meeting is centred around opportunities (O): In the first week of NICU admission, consultation regarding the wishes and needs of parents are explored and discussed (by a neonatal nurse and social worker). The third meeting is about integration (I): During the NICU admission period, the focus is on integration of the parents' wishes and needs, including their involvement in care and treatment of their infant (by neonatal nurse, physiotherapists, and occupational therapists). This meeting is repeated on a weekly basis till the last week of the expected discharge. The fourth meeting is held in the week of expected discharge and is focused on control (C): At the end of the NICU admission, the focus is on the knowledge of parents regarding the transition to discharge and the care at home (by neonatal nurse). The final meeting is evaluation (E): During the follow‐up visit (6 weeks after term date), the focus is on evaluating the experiences of the parents regarding the NICU admission and discussing emerging questions regarding care of their infant in their home setting (by social worker and neonatal nurse).

In addition to these series of VOICE meetings, parents are invited to visit the weekly parental classroom meetings (Figure 1). During these meetings, parents are educated by physiotherapists, occupational therapists, lactation specialists, and nurses on several subjects such as breastfeeding, developmental care, learning to know your infant, and transition to another department or another hospital. In these weekly parental meetings, an exchange of thoughts and feelings of parents is discussed, with emphasis on positive support for the parents.

### 2.5 Data collection

*S*emi‐structured interviews were conducted between February 2017 and October 2017. The interviews were audio‐taped and transcribed ad verbatim. Interviews were conducted in Dutch and held in a quiet room in the hospital prior to discharge of the infant. Because of the nature of data collection in this study, by organizing the interviews before discharge, we were unable to evaluate the E (Evaluation meeting at follow up) of the VOICE programme. However, this evaluation will be performed at the follow‐up clinic in another study. The researcher (MJE) who performed the interviews was trained in interview techniques, and pilot interviews were performed.^14^ The interview guide was based on recent literature and expert opinion (Electronic Supplement Material 1). Demographic characteristics of the study participants, such as gender, age, education level of the parents and GA, birthweight, and admission time of the infants were collected during the interviews. Data were collected until data saturation was reached. Data saturation means that no new information is collected regarding the selected research topic.

### 2.6 Data analysis

A thematic analysis was performed using an inductive approach; data coding was performed without using a pre‐existing framework. Thematic analysis involves searching and coding across a data set to find repeated patterns of meaning, so‐called themes. We adopted the thematic analysis described by Braun and Clarke.^18^ This involves six phases to explore meaningful repeated patterns in the data: Step 1 was familiarizing with the data—the manuscripts were read several times. To ensure rigour and trustworthiness, each transcript was read and coded separately by two researchers (ME and FJ) independently. In step 2, generating initial codes, the individual narratives of parents were coded. Step 3, searching for themes, was performed by collating codes into sub‐themes. If uncertainty appeared in this process, the linked narratives belonging to the codes were reviewed to better understand the underlying meaning of the code and the sub‐theme. Step 4, reviewing themes, was performed by combining sub‐themes with themes if appropriate. If sub‐themes were clearly indicating a specific meaningful theme, this was retained as an individual theme. Step 5, defining and naming themes, was the ongoing analysis of reviewing the codes and generated (sub‐)themes. Refinement of the themes was considered to improve the clarity and relevance of the themes. This process was performed with a third researcher (AvdH). Any disagreement of the codes, sub‐themes, and themes was solved by discussion. For step 6, producing the report, the findings are reported in this paper.

Consensus among the researchers was reached after each step.

Furthermore, member checking was performed by sending the participating parents a summary of the analysis, asking if they agreed with the findings and if there were missing determinants. The used quotations in the results section are anonymized by codes: M = mother and F = father along with the number of the study interview.

### 2.7 Ethical considerations

Verbal and written information was provided to eligible parents. All participating parents provided written informed consent. The Medical Research Ethics Committee of the University Medical Centre Utrecht approved the study (Protocol number: 17‐059/C).

## 3 FINDINGS

Eleven semi‐structured interviews with parents were completed with 13 participants: nine interviews with mothers and two interviews with both mothers and fathers (Table 1). The interviews lasted between 30 and 45 minutes. Mean age of the parents was 33 years (range 28‐43). GA of the infants was between 24 and 27 (mean 25.8) weeks and birthweight between 700 and 1070 (mean 899) grams (Table 1).

**TABLE 1:** Characteristics of parents (n = 13) and infants (n = 11)

Interviews mother (M) father (F)	Parents age	Parent education h = high m = moderate	Siblings No/Yes	Infant gender (M/F)	Infant GA (weeks)	Infant birthweight (gr)	Infant LoS NICU (days)
M1/F1	30/30	h/h	No	F	26^5/7^	955	80
M2/F2	28/30	h/h	No	M	25^4/7^	930	121
M3	38	m	Yes	M	26	950	128
M4	43	h	Yes	M	25	710	94
M5	34	h	No	M	24^3/7^	700	126
M6	30	h	No	M	26^6/7^	1025	43
M7	37	h	Yes	M	26^1/7^	900	43
M8	33	h	No	M	26^5/7^	1030	40
M9	35	m	Yes	M	26^4/7^	800	104
M10	28	h	No	F	24	710	79
M11	34	h	No	F	26^1/7^	1070	105

Abbreviations: gr = grams; LoS = length of stay; M/F = male/female.

The findings have been described in one overarching theme: “parental empowerment” (Table 2). Parents felt strengthened and were empowered by their involvement in care and the VOICE programme. This is reflected by four related interpretive themes: (a) involvement in care, (b) personalized information and communication, (c) transition to a parental role, and (d) emotional support (Table 2).

**TABLE 2:** Summary of overarching and interpretive themes and quotations

Overarching theme	Interpretive themes	Quotations
Parental empowerment	1. Involvement in care	Involved as a partner in health care by caring myself for my baby made me strong (M8)
		Involved in care gave confidence (F2)
		The webcam made it possible to be involved and it helped me even while pumping breastmilk (M10)
		The possibility to be more connected by caring is appreciated very much (M6)
	2. Personalized information and communication	Parental meetings were very informative in education about the principles of developmental care (M5)
		Other parents asked questions, which contributed to knowledge (M5)
		Motivating to get information and to learn to observe the behaviour of my infant together (M6)
		A huge amount of written and oral information during NICU admission and difficult to remember all of it (M9)
	3. Transition to a parental role	We did the care all by ourselves. It was our own process and very meaningful for us to feel complete as a parent (M5)
		Being involved as a partner in health care contributes to my parental feelings (M8)
		As a father, I have the full responsibility for my infant. In order to fulfill my role as a father, I need to know about all the daily choices and considerations of the doctors (F1)
		Information about handling and positioning are not only useful in the NICU period but also in the period after admission (M5)
	4. Emotional support	Nurses and doctors were very friendly and always asking how we were doing, this was very supporting (M9)
		We were surviving in the NICU and without the social worker we hadn't discussed feelings of mourning and anxiety. It helped us to reflect on our situation (M5)
		It was nice to tell my story to the nurses and to have somebody who was just listening and who understood the situation on the NICU (M9)
		We had a lot of contact with our relatives but everyone liked to hear good news. It helped to talk with other parents from the NICU (F1)

Abbreviation: NICU, neonatal intensive care unit.

### 3.1 Involvement in care

The VOICE meetings helped parents express their needs and wishes and how they wished to participate in caring for their infant in a way that they wanted. Parents valued active participation in the care of their infants and having skin‐to‐skin contact stimulated. It empowered the parental role: “To hold him and to care for him gave me warm feelings and contributed to stronger feelings of being a mother” (M7). The possibilities of being present 24/7 at the ward and to be engaged in caring for the infant were very important for parents. In addition, gaining confidence through the NICU staff during the VOICE meetings and being connected are important for parents: “A very experienced nurse, involved us in care of our infant and gave us a lot of confidence” (F2). In summary, participation in the VOICE programme and involvement in care are important factors to support and empower parents in the NICU.

### 3.2 Personalized information and communication

All parents indicated that personalized, open, understandable, and honest communication from the NICU staff throughout the VOICE meetings was very important to facilitate a positive parent‐staff relationship. Important areas of information and support would include information on infant health, both medical and technical; infants' care; and how to be involved. Neonatologists and neonatal nurses are the primary sources of information. Parents appreciated it when the same doctor and nurses were responsible for their infant during the NICU admission. The nurses, physiotherapists, and occupational therapists informed and educated the parents about the principles of developmental care, as one mother mentioned: “The NICU nurse encouraged us to participate in the care. We learned a lot by observing how nurses cared and by copying their practice” (M9). Parents who attended the educational parental meetings reported that this programme gave them much information and support from other participating parents: “The parental meetings were very informative and it was very nice to meet other NICU parents to share some feelings and experiences about behavioural cues and because other parents asked questions, which contributed to my knowledge” (M5). In addition, parents were positive about the VOICE meetings and the personalized training with the physiotherapist or occupational therapist, where they received information to observe their infant's behaviour when caring for their infant, like one mother said “It was very motivating to get information and to learn to observe the behaviour of my infant together. I looked forward to the next round to handle even more sensitive than I already did” (M6). Overall, parents feel empowered in their parental role when they are informed and encouraged to participate in care and decision‐making. The VOICE programme contributes to their knowledge.

### 3.3 Transition to a parental role

Parents indicated that the VOICE programme changed their role as a parent from feeling powerless and “can't do anything” to fully participating in their infants' care and decisions. Parents indicated that it was very important to obtain control over the care of their infants in order to establish their role as parents. They need confidence to do so, as some mothers indicated: “We did the care all by ourselves. It was our own process and very meaningful for us to feel complete as a parent” (M5). Other parents mentioned that they felt it was their responsibility to be involved in the health care team as a serious partner. “As a father, I have the full responsibility for my infant. In order to fulfill my role as a father, I need to know about all the daily choices and considerations of the doctors” (F1). Briefly, the VOICE meetings contribute to participating in the care and empowerment of parents. In addition, the program also helps to support and accept their parental role.

### 3.4 Emotional support

Emotional support by neonatal staff was important throughout the VOICE meetings: “The nurses were so very friendly, and kind and the doctor always asked how we were doing, this was very supporting” (M9). Most of the parents experienced the VOICE meetings as valuable in supporting their emotional feelings. Parents mentioned that individual emotional support and confirmation of what they did well was of great value: “We got a lot of compliments and it supports us to feel positive and to feel more confident with the whole situation” (M9). Parents made a distinction between the practical information they received from the social worker and emotional support from others. Practical information such as how to deal with the duration of maternity leave and the possibilities of postponed maternity care was given to all parents and reiterated during the C meeting (control) in the VOICE programme. All parents expressed that this kind of practical information was very useful. Emotional support targeted the emotional rollercoaster parents faced in the NICU. This was often discussed in the O (opportunities) and I (integration) meetings of the VOICE programme with various team members attending, like one mother mentioned: “We were surviving in the NICU and without the social worker we hadn't discussed feelings of mourning and anxiety. It helped us to reflect on our situation” (M5). Some parents indicated that they had no need to share emotional feelings during the VOICE meetings. They preferred to discuss emotions with their partner and other relatives. Other parents indicated that sharing their story and feelings helped them to process all the things that happened around the birth and admission to the NICU: “It was nice to tell them my story and to have somebody who was just listening and who understood the situation on the NICU” (M9).

The VOICE meetings have been supporting the parents specifically regarding the feelings of being on an “emotional rollercoaster.” However, some parents also want to share their thoughts and emotions with peers.

## 4 DISCUSSION

The findings of our study regarding the experiences of parents participating in the VOICE programme during NICU admission identified one overarching theme: “parental empowerment.” Empowerment reflects on knowledge, capabilities, motivation, and opportunities.^14^ It is a process, however, and there is no unambiguous definition. Instead, a variety of definitions is known, and often, empowerment refers to a combination of ability, motivation, and increased opportunities, including activation, enablement, involvement, and participation.^19^ Parents indicated that the VOICE meetings empowered them and helped them to gain more knowledge and experiences in caring for their infant, which improved their parental role. Our findings highlight the need for support and promote the application of the principles of FCC.^20^

All parents indicated that personalized, open, understandable, and honest communication in receiving information from NICU staff was very important to facilitate a positive parent‐staff relationship. This confirms the results of the study by Friedman et al, who showed that a collaborative open interaction with the neonatal staff is an important factor for parents to feel comfortable in NICU settings.^21^, ^22^ Parents are supported to discuss their involvement in their infants' care, with increasing responsibility during admission till discharge. Support and personal information are important in making parents feel valued and become active partners. As documented in the literature and in our study, neonatal nurses have an important role in guiding parents to become comfortable and autonomous.^21^, ^23^ Previous research emphasized that giving parents the opportunity to perform care routines by themselves and supervising them in a positive way improves the parent‐infant relationship, as well as the parent‐nurse relationship.^24^, ^25^

Parents were positive about the individual support received during the VOICE meetings, which contributed to a higher sensitivity and better understanding of their own and infant's needs. A positive approach to meet the individual needs of parents provides confidence in the day‐to‐day care.^24^ Parent participation in educational programmes providing information and opportunities for sharing has been shown to reduce parental stress and anxiety and improves confidence and competence.^12^, ^23^, ^26^ This corresponds to the findings of our study where parents gain more insight in how they could support their infants in an optimal way, which empowered them. To increase learning and to meet the needs of parents, studies have indicated that the use of multiple approaches is important.^8^, ^27^ Different educational programmes have been demonstrated that a combination of observation, written information, and discussions is the preferred method to support parents.^26^ This is also shown in our VOICE programme, where parents receive information and education during the VOICE meetings and the weekly parental educational sessions with experts. In addition, parents receive medical and technical information from doctors and nurses during daily rounds, where parents are invited, which they valued as very important.

Providing support to parents is one of the key caring responsibilities of NICU staff, specifically in connection with the family‐centred care approach. The VOICE programme was initiated to provide a structured approach to support parents throughout the pathway of a NICU admission. The programme was initiated to provide structured support to complement other support that is often provided in unscheduled conversations at the bedside. We acknowledge that our VOICE programme complements other interventions to support parents, which have been standard practice for some years in the NICU community. An example is the intervention related to new mothers who received peer support through a “buddy” programme. These mothers experienced less anxiety and greater social support than mothers who did not participate in the buddy programme.^23^, ^28^ Perhaps the synergy of various support programmes in a NICU can contribute to the empowerment and partnership between parents and staff; the whole is greater than the sum of the parts.

In our study, the VOICE programme corresponds with many aspects of FCC in the NICU and therefore might be considered a transferable and beneficial programme in neonatal care.^8^, ^29^ Understanding the needs of parents, to empower them and to give them confidence, is an important goal of the VOICE programme. Actively listening to the views of parents is a powerful element to understand the individual needs and to create a fundamental improvement in the quality of care based on empowerment of parents. In order to empower parents and support them in their parental role to reduce stress and anxiety before, during, and after NICU admission, parents need to be involved as partners in care in every neonatal ward and NICU globally. However, “parental empowerment interventions” in the NICU need more robust studies to confirm the effectiveness on parents' health outcomes and infants' clinical outcomes.^12^

### 4.1 Strength and limitations

The strengths of our study were that the newly introduced VOICE programme was evaluated by interviewing parents (both mothers and fathers) who were involved in the programme. Another strength was the rigour and trustworthiness of the qualitative methods by training junior researchers, involving experienced qualitative researchers in the analysis and constant feedback. Limitations were the origin of the different parents included. The participants were mostly Caucasian Dutch mothers, and only two fathers participated. Parents of other ethnicities might have different experiences and needs. Future studies need to confirm the impact of these cultural differences. Another limitation could be the small sample size; however, after 10 interviews, saturation of data was reached, and no new information was gained. Therefore, after the 11th interview, the recruitment was stopped. Finally, the VOICE programme was evaluated with parents who were still present at the NICU. The conversations of the fifth VOICE meeting (evaluation) have not been explored. Further studies should test the full programme, including long‐term follow up.

## 5 CONCLUSION

Participation and involvement in care with personalized structured and focussed meetings are important initiatives to support parents in the NICU. There is a need for transparent, clear, and respectful communication between parents and health care professionals. A multidisciplinary approach adds value to supporting parents in their role in the NICU. The VOICE programme is a structured framework of implementing family support in the NICU to support and empower parents. Further studies are needed to confirm the effect on parental outcomes and infants' health outcomes.

## AUTHOR CONTRIBUTIONS

**Agnes van den Hoogen**, **Rian Eijsermans**, and **Jos M. Latour:** Designed the study and protocol. **Henriette D. L. Ockhuijsen**, **Floor Jenken**, **Sabine M. Oude Maatman**, **Marian J. Jongmans**, **Lianne Verhage**, **Janjaap van der Net:** Provided support to the study team. **Rian Eijsermans** and **Agnes van den Hoogen:** Contributed to the data collection. **Rian Eijsermans**, **Agnes van den Hoogen**, **Henriette D. L. Ockhuijsen**, **Floor Jenken,** and **Jos M. Latour:** Performed data analysis and interpretation. **Rian Eijsermans** and **Agnes van den Hoogen:** Drafted the first manuscript. All authors provided comments and approved the final manuscript.

## ETHICS STATEMENT

The protocol was approved by the Ethics Committee of the University Medical Centre of Utrecht, Protocol number 17‐059/C. Parents were informed that their decision to refuse or withdraw from the study would not impact the care of their infant. All procedures performed in the studies were in accordance with the Declaration of Helsinki.

## PATIENT CONSENT STATEMENT

Written informed consent was obtained from all parents included in the study.

## PERMISSION TO REPRODUCE MATERIAL FROM OTHER SOURCES

All material from other sources are referenced in the manuscript.

## Supporting information

Electronic Supplement Material 1: Interview guide

## Data Availability

The authors confirm that the data supporting the findings of this study are available within the article [and/or] its supplementary materials.
