# Supplementary material for: Parents' experiences of VOICE: A novel support programme in the NICU
Source: Nurs Crit Care. 2020 Oct 29;26(3):201–8. doi: 10.1111/nicc.12569 (PMC8246858; doi:10.1111/nicc.12569)
Supplement: Supplementary file 1 — Electronic Supplement Material 1: Interview guide [file NICC-26-201-s001.docx]

**Electronic Supplement Material 1: Interview guide**

General questions

• What was your first impression when you arrived at the NICU?

• How did you experienced the first week of admission at the NICU as a parent?

• How did you experience your parental role during admission?

• What did you need as a parent to grow into your parental role?

• How can health professionals help you to support you?

VOICE meetings and parental classes

• How did you experience the VOICE programme and meetings?

• How did these meetings contribute to your knowledge and parental role?

• How did you experience the contact with other parents during the classes?

• Do you have suggestions for other topics to discuss with parents during the parental classes?
